# Antioxidant action of six *Trifolium* species in blood platelet experimental system in vitro

**DOI:** 10.1007/s11010-015-2556-2

**Published:** 2015-09-08

**Authors:** J. Kolodziejczyk-Czepas, P. Nowak, I. Kowalska, A. Stochmal

**Affiliations:** Department of General Biochemistry, Faculty of Biology and Environmental Protection, University of Lodz, Pomorska 141/143, 90-236 Lodz, Poland; Department of Biochemistry, Institute of Soil Science and Plant Cultivation, State Research Institute, Czartoryskich 8, 24-100 Pulawy, Poland

**Keywords:** Antioxidant, Blood platelets, Clover, Oxidative stress, *Trifolium*

## Abstract

This study includes a comparative evaluation of antioxidant effects of plant extracts (1.5–50.0 μg/ml), derived from six clover (*Trifolium*) species: *T. alexandrinum* L., *T. fragiferum* L., *T. hybridum* L., *T. incarnatum* L., *T. resupinatum* var. *majus* Boiss., and *T. resupinatum* var. *resupinatum* L. Chemical profiles of the extracts contained three or four groups of (poly)phenolic compounds such as phenolic acids, clovamides, isoflavones, and other flavonoids. Antioxidant properties of *Trifolium* extracts were assessed as the efficacy to reduce oxidative and nitrative damage to blood platelets, exposed to 100 μM peroxynitrite-induced oxidative stress in vitro. Antioxidant actions of the examined extracts were determined by the following biomarkers of oxidative stress: thiol groups, 3-nitrotyrosine, lipid hydroperoxides, and thiobarbituric acid-reactive substances (TBARS). Despite the significant differences in the chemical composition (the total phenolic concentrations varied between 11.30 and 52.55 mg/g of dry mass) of *Trifolium* extracts, we observed noticeable protective effects of almost all tested plant preparations. The *T. alexandrinum* extract, containing the highest concentration of phenols, was the most effective antioxidant among the tested extracts. On the other hand, the *T. incarnatum* extract, which contained a comparable total phenolic content (49.77 mg/g), was less efficient in prevention of tyrosine nitration and generation of TBARS. These findings indicate on the important role of individual phenolic components of the examined clover extracts for the final antioxidative effects. Antioxidative properties of the remaining extracts were noticeably weaker.

## Introduction

Inflammation, oxidative stress, activation of blood platelets, and coagulation cascade are strictly linked and may result in pathophysiological consequences, including thromboembolic complications [1]. For that reasons, the scientific interest in new substances displaying antioxidative properties (plant-derived compounds, in particular) has been growing. Clovers (*Trifolium*, Leguminosae) have been used in traditional medicine by various cultures, and several species are grown as pasture crops for animals [2]. However, the majority of scientific reports concerning the biological activity of *Trifolium* species contain results of in vitro and in vivo studies on *T. pratense* (red clover). The traditional medicine recommendations of this plant include its use as expectorant, antiseptic and analgesic remedy as well as administration for sore throat, fever, pneumonia, and meningitis; skin problems, lung illnesses disorders of reproductive system [3]. Currently, red clover is a source of numerous dietary supplements with phytoestrogenic effects, consuming as alternatives to estrogen replacement therapy [4]. Pharmacological effects of clovers other than *T. pratense* are less known; however, some encouraging information is available [5–7]. Similarly, the influence of *Trifolium* species on the cardiovascular system has been only partly recognized. Antioxidant properties of *T. pratense* have been recently confirmed by Vlaisavljevic et al. [8]. On the other hand, results of the pilot clinical trial, described by Campbell et al. [9], revealed little or no effect of isoflavone supplementation (86 mg/day of red clover-derived isoflavones for 1 month) on antioxidant status, whereas results obtained by Asgary et al. [10] indicated on the cardiovascular disease-preventive properties of red clover. The cited studies, performed on the animal model of atherosclerosis, demonstrated a significant decrease of C-reactive protein, triglyceride, total cholesterol, and LDL-cholesterol levels, whereas HDL-cholesterol level was increased. Beneficial effects of *Trifolium pratense*-derived isoflavones on the lipid profile of postmenopausal women with increased body mass index, have been also shown [11]. However, it should be emphasized that the beneficial influence of red clover and other clovers may be dependent not only on the content of isoflavones, but also on actions of numerous bioactive substances occurring in these herbs.

The present comparative study includes the evaluation of antioxidative activities of extracts obtained from 6 clover species: *T. alexandrinum* L., *T. fragiferum* L., *T. hybridum* L., *T. incarnatum* L., *T. resupinatum* var. *majus* Boiss., and *T.resupinatum* var. *resupinatum* L. Antioxidant actions of the extracts were examined in an experimental system of blood platelets, exposed to peroxynitrite-induced oxidative stress in vitro. The species were chosen on the basis of our previous examination of phytochemical profile [12], agricultural significance as well as existing evidence of their biological activities. Clovers such as *T. pratense* L., *T. resupinatum* L., *T. incarnatum* L., *T. hybridum* L., and *T. fragiferum* L. are well known forage plants, but some data on the possible therapeutic effects have been reported. For instance, besides phytoestrogenic properties of *T. pratense*, traditional medicine uses of this plant include also expectorant, antiseptic, analgesic, sedative, and tonic mixtures. Seeds of *T. alexandrinum* L. have been recommended in Egypt as an anti-diabetic remedy, whereas contemporary studies on this plant have demonstrated hepatoprotective [13] and antibacterial [14] action of preparations from aerial parts of this clover. Furthermore, Budzynska et al. [15] reported recently antimicrobial activity of saponin-rich fractions, isolated from aerial parts of *T. alexandrinum*, *T. incarnatum* and *T. resupinatum* var. *resupinatum*. The anti-inflammatory and antioxidative properties of *T. resupinatum* were also described in the literature [16].

## Materials and methods

### Chemicals

Anti-3-nitrotyrosine polyclonal antibody, biotin-conjugated secondary antibody, and Strept/HRP were obtained from Abcam (Cambridge, UK). Peroxynitrite was synthesized according to the method of Pryor et al. [17]. Fibrinogen for a competitive ELISA test was prepared from human blood according to the method described by Doolittle’ et al. [18]. 5,5′-dithiobis-(2-nitrobenzoic acid) (DTNB, Ellman’s reagent), thiobarbituric acid, trichloroactetic acid, and Sigma OPD Fast Substrate were purchased from Sigma-Aldrich. All other reagents were of analytical grade and were provided by commercial suppliers.

### Plant material

The aerial parts of six tested *Trifolium* species were grown and harvested at the Station of Vegetation Experiments of the Institute of Soil Science and Plant Cultivation - State Research Institute in Pulawy (Krolewska st17, Poland) [19]. Phenolic fractions of *Trifolium* species were isolated with the method previously described for *Medicago sativa* L. (Fabaceae) [20]. Briefly, the freeze dried and powdered plant material was extracted in 40 % methanol (v/v) at room temperature for 24 h. The extracts were filtered, and then, the supernatants were evaporated, dissolved in distilled water and fractionated by the low-pressure liquid chromatography, with the use of a reversed phase (RP) C18 column (60 × 100 mm, 40–60 μm, Merck). First, the column was washed with water in order to remove saccharides, and then, 40 % methanol (v/v) was used as the eluent of phenolic fraction. Profiles of phenolic fractions obtained in this way were qualitatively and quantitatively analyzed by using the ultra performance liquid chromatography (UPLC) [19].

The examined extract differed substantially in regard to the total content of phenolic substances. *T. alexandrinum* and *T. incarnatum* contained significantly higher concentrations of phenols 52.55 and 47.97 mg/g of dry mass, respectively. The total contents of phenolics in *T. fragiferum*, *T. hybridum*, *T. resupinatum* var. *majus*, and *T. resupinatum* var. *resupinatum* were 11.30, 15.24, 22.54, and 17.32 mg/g of dry mass, respectively. Concentrations of individual groups of phenolic compounds are presented in Table 1 [19].

**Table 1:** Contents (mg/g of dry mass) of phenolic acids, flavonoids, isoflavones, and clovamides, determined in the examined *Trifolium* extracts (phenolic fractions, obtained from aerial parts of the plants) [19]

Groups of phenolic compounds	*Trifolium* species					
	*T. alexandrinum*	*T. fragiferum*	*T. hybridum*	*T. incarnatum*	*T. resupinatum* var. *majus*	*T. resupinatum* var. *resupinatum*
Phenolic acids	1.65	0.62	9.58	1.32	1.61	0.86
Clovamides	9.63	–	0.44	–	–	–
Isoflavones	18.97	5.50	–	5.10	11.17	5.21
Flavonoids	22.30	5.18	5.22	41.54	9.76	11.25

### Isolation of blood platelets

Blood samples from healthy volunteers were purchased from the Regional Centre of Blood Donation and Blood Treatment in Lodz, Poland. Platelets were sedimented by centrifugation, performed accordingly to protocol of Wachowicz and Kustron [21]. Platelet pellet was suspended in Tyrode’s buffer (10 mM HEPES, 140 mM NaCl, 3 mM KCl, 0.5 mM MgCl_2_, 5 mM NaHCO_3_, 10 mM glucose, pH 7.4). In platelet suspensions, used for experiments, the amount of platelets was about 5 × 10^8^/ml (as was estimated spectrophotometrically [22] ).

### Samples preparation

Stock solutions of the tested extracts were made in 25 % dimethylsulfoxide (DMSO); effects of the solvent were excluded. Suspensions of platelets were pre-incubated for 10 min at 37 °C with the *Trifolium species*-derived extracts, added to the final concentration range of 1.5–50.0 µg/ml. Samples were then exposed to 100 µM peroxynitrite (ONOO^−^). To assess antioxidative effects of the extract, also platelet suspensions treated with peroxynitrite in the absence of the extracts were analyzed. As a reference compound, 12.5 µM (−)-epicatechin was used. The control samples contained platelets untreated with the extracts and/or peroxynitrite. In some experiments, blood plasma (diluted with 0.1 M Tris/HCl, pH 7.4, buffer to a concentration of 2 mg/ml) treated with peroxynitrite in the presence/absence of extracts (or (−)-epicatechin) was used.

### Determination of 3-nitrotyrosine in the proteins of human platelets the competitive ELISA test

The detection of 3-nitrotyrosine in blood platelets was performed according to the modified method of Khan et al. [23], as described by Olas et al. [24]. The concentrations of nitrated proteins were estimated from the standard curve as 3-nitro-fibrinogen (3-NT-Fg) equivalents.

### Determination of thiols

Thiol groups in blood platelet proteins were determined by using 5,5′-dithio-bis(2-nitro-benzoic acid) (DTNB) [25].

### Measurements of lipid peroxidation biomarkers

The peroxynitrite-induced lipid peroxidation in blood platelets was determined colorimetrically, by measurements of two oxidative stress biomarkers: lipid hydroperoxides (estimated by the ferric-xylenol orange (FOX-1) assay Gay and Gebicki [26]) and thiobarbituric acid-reactive substances (TBARS) [27].

### Immunodetection of 3-nitrotyrosine by Western blot (WB) analysis

Antioxidant effects of the examined *Trifolium* extracts were additionally confirmed by the WB technique. For these experiments, one concentration (12.5 μg/ml) of both the extracts and (−)-epicatechin was chosen. The samples of platelets or blood plasma proteins were separated by the SDS-PAGE method [28] and transferred to Immobilon P membrane. 3-NT-containig proteins were detected by incubation of the blots with anti-3-NT antibody and horseradish peroxidase-coupled secondary antibody. Results were visualized with a chemiluminescence kit and recorded on the Roentgen film.

### Data analysis

Uncertain data were excluded by the Q-Dixon test. All the values in this study were expressed as mean ± SD. Statistical significances were evaluated by the t-Student’s test as well as by ANOVA and the Dunnett test.

## Results

**Fig. 1:**
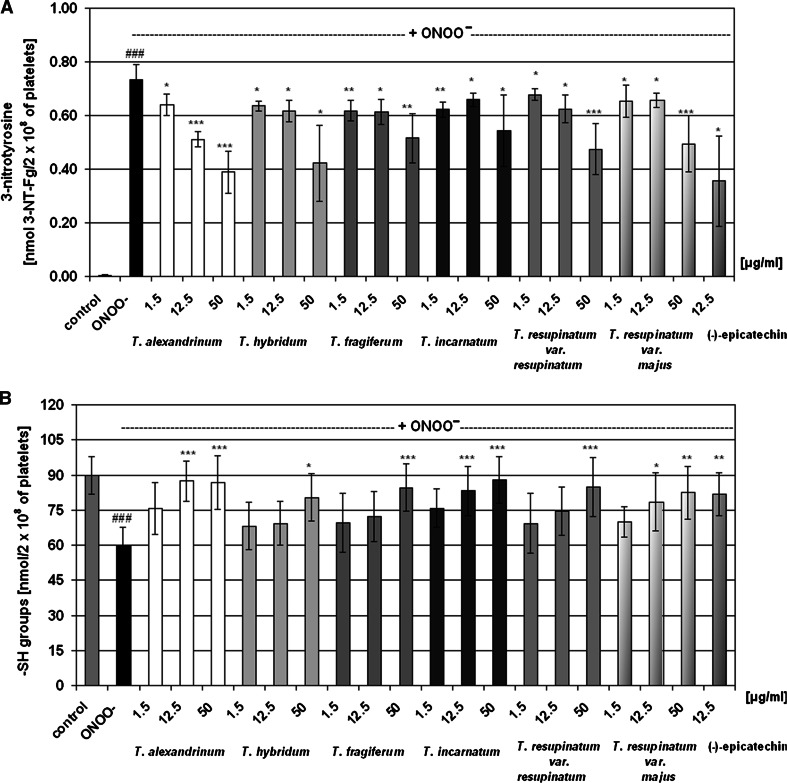
Protective effects of *Trifolium* extracts on peroxynitrite-induced damage to blood platelet proteins. The anti-nitrative action of the extracts (**a**) was assessed by the c-ELISA test, a semi-quantitive method, used for the measurements of 3-NT level. Oxidation of thiol groups (**b**) was determined with the use of Ellman’s reagent. Results are presented as mean ± SD; control platelets versus ONOO^−^-treated platelets (without the extracts): ^###^ *p* < 0.001; ONOO^−^-treated platelets in the absence of the extracts versus ONOO^−^-treated platelets in the presence of the extracts: *p < 0.05, **p < 0.01, ***p < 0.001; *n* = 6

**Fig. 2:**
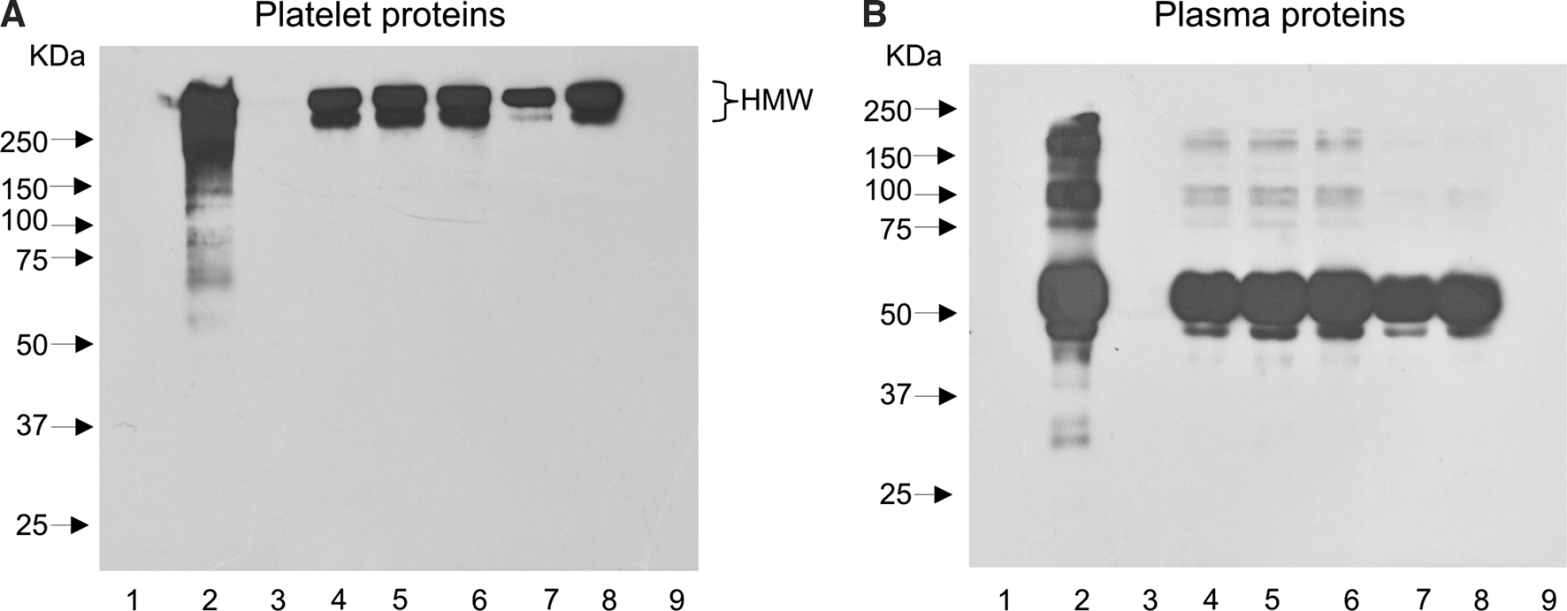
Comparison of antioxidant actions of the examined *Trifolium* extracts on ONOO^−^-induced tyrosine nitration in blood platelet (**a**) and plasma (**b**) proteins. The picture represents WB patterns of 3-nitrotyrosine-containing proteins. Blood plasma and platelet proteins were separated by SDS-PAGE method (in 7.5 % gels) and transferred to Immobilon P membrane. The immunodetection of 3-NT was performed with the anti-3NT antibody, and then, the results were visualized with a chemiluminescence kit. Platelet proteins were separated under non-reducing conditions, whereas samples of plasma proteins were analyzed under reducing conditions. Approximately 10 µg of protein was applied to each lane. Molecular weight markers are indicated on the *left*. Representative blots of three independent experiments are shown. *Lane 1* control sample, *lane 2* peroxynitrite-treated samples (without the extracts), *lanes 3–8* correspond to samples of platelets (**a**) or plasma (**b**) preincubated with *Trifolium* extracts (12.5 μg/ml) and exposed to 100 μM ONOO^−^ (lane 3 *T. alexandrinum*, lane 4 *T. hybridum*, *lane 5* *T. fragiferum*, lane 6 *T. incarnatum*, lane 7 *T*. *resupinatum* var. *resupinatum*, lane 8 *T. resupinatum* var. *majus*). *Lane 8* represents samples preincubated with (−)-epicatechin (12.5 μg/ml) and exposed to peroxynitrite

Analysis of oxidative stress biomarkers revealed oxidative alterations in both protein and lipid components of blood platelets, induced by exposure to 100 μM peroxynitrite. The level of platelet thiols was reduced by about 30 %, whereas results of immunodetections by c-ELISA test demonstrated an evident increase of 3-NT content in platelet proteins. In samples treated with peroxynitrite in the presence of *Trifolium* extracts (at concentrations of 1.5–50.0 µg/ml), the extent of oxidative and nitrative damage to platelet proteins was significantly decreased. The obtained results were compared with action of (−)-epicatechin, a well-known polyphenolic antioxidant (Fig. 1), displaying a unique ability to prevent nitration reactions, mediated by peroxynitrite [29]. Statistically significant reduction of tyrosine nitration by the extracts was observed for all the tested *Trifolium* species; however, the anti-nitrative action of *T. alexandrinum* was visibly stronger (Fig. 1a). This observation was confirmed by the Western blotting method, during additional immunodetections of 3-NT, conducted on blood platelets (Fig. 2a) and plasma (Fig. 2b). The WB immunodetections of 3-NT in blood plasma exposed to ONOO^−^ demonstrated several bands corresponding to blood plasma proteins with molecular weight of 20–230 kDa, whereas in samples treated with the oxidant in the presence of *T. fragiferum, T. hybridum, T. incarnatum, T. resupinatum* var. *resupinatum, and T. resupinatum* var. *majus* extracts, primarily two bands (corresponding to proteins with molecular weight about 50–65 kDa) were found. In the WB patterns of blood plasma pre-incubated with 12.5 µg/ml of *T. alexandrinum* extract or (−)-epicatechin, and exposed to 100 µM ONOO^−^ no chemiluminence was recorded (Fig. 2b). Analysis of the WB patterns of blood platelets indicates on ONOO^−^-induced formation of protein aggregates, with molecular weight over 250 kDa. The lack of chemiluminence in lanes corresponding *T. alexandrinum* extract and (−)-epicatechin samples confirmed strong ONOO^−^-scavenging activities of these substances (Fig. 2a). Similarly, measurements of thiol group concentrations also indicated *T. alexandrinum* extracts as the most efficient antioxidant. The content of thiol groups in samples pre-incubated with this extract at concentrations of 12.5 and 50.0 μg/ml was comparable to control platelets (*p* > 0.05). Furthermore, comparison of antioxidant effects *T. alexandrinum* extract and (−)-epicatechin (at the same concentration of 12.5 μg/ml) revealed that the extract was more effective (Fig. 1b). On the contrary, antioxidative activities of *T. hybridum* and *T. fragiferum* in the protection of platelet proteins were noticeably weaker than *T. alexandrinum*, both in WB immunodetections of 3-NT (Fig. 2), as well as during colorimetric evaluation of the –SH groups level (Fig. 1).

Antioxidant effects of the examined extracts were also determined by using lipid peroxidation biomarkers (Table 1). Results of the FOX-1 assays revealed that all of the tested extracts were able to diminish generation of lipid hydroperoxides, but anti-lipoperoxidative action of *T. resupinatum* var. *resupinatum* was slightly weaker. Measurements of TBARS confirmed evidently stronger antioxidant properties of *T. alexandrinum*, whereas *T. fragiferum* (*p* > 0.05 for all concentrations) and *T. resupinatum* var. *resupinatum* (*p* > 0.05 for concentrations of 1.5–12.5 μg/ml) extracts were less effective antioxidants (Table 2).

**Table 2:** The protective effects of phenolic fractions from six *Trifolium* species on ONOO^−^-induced oxidative damage to lipid component of blood platelets

	Extract or (−)-epicatechin (µg/ml)	Lipid hydroperoxides (nmol/2 × 10^8^ platelets)	Thiobarbituric acid-reactive substances (TBARS) (nmol/2 × 10^8^ platelets)
Control platelets (untreated)	0	1.238 ± 0.085	0.484 ± 0.036
Platelets exposed to ONOO^−^ in the absence of any extract	0	5.665 ± 1.472^###^	0.864 ± 0.098^###^
Platelets exposed to ONOO^−^ in the presence of the examined extracts or (−)-epicatechin			
* T. alexandrinum*	1.5	3.659 ± 0.738***	0.620 ± 0.096***
	12.5	3.465 ± 0.884***	0.615 ± 0.059***
	50.0	3.906 ± 0.666**	0.571 ± 0.060***
* T. hybridum*	1.5	3.576 ± 0.850**	0.776 ± 0.108
	12.5	3.703 ± 0.677**	0.701 ± 0.055^**^
	50.0	3.499 ± 0.598**	0.673 ± 0.078^**^
* T. fragiferum*	1.5	3.863 ± 0.606**	0.853 ± 0.082
	12.5	3.640 ± 0.849***	0.855 ± 0.088
	50.0	3.734 ± 0.813***	0.738 ± 0.093
* T. incarnatum*	1.5	3.630 ± 0.686***	0.758 ± 0.095***
	12.5	3.637 ± 0.954***	0.681 ± 0.103***
	50.0	3.708 ± 0.862***	0.620 ± 0.088***
* T. resupinatum* var. *resupinatum*	1.5	4.699 ± 0.742	0.824 ± 0.087
	12.5	4.023 ± 0.968*	0.802 ± 0.072
	50.0	3.398 ± 0.959**	0.714 ± 0.040*
* T. resupinatum* var. *majus*	1.5	3.364 ± 0.861***	0.765 ± 0.090
	12.5	3.539 ± 0.996***	0.679 ± 0.070***
	50.0	3.659 ± 0.973***	0.598 ± 0.094***
(−)-epicatechin	12.5	2.636 ± 1.030***	0.611 ± 0.048***

The anti-lipoperoxidative actions of *Trifolium* extracts were estimated with the FOX-1 reagent and as the generation of TBARS. Results are presented as mean ± SD; control platelets versus ONOO^−^-treated platelets (without the extracts): ^###^ *p* < 0.001; ONOO^−^-treated platelets in the absence of the extracts versus ONOO^−^-treated platelets in the presence of the extracts: *p < 0.05, **p < 0.01, ***p < 0.001; *n* = 7

## Discussion

In the present study, the experimental model of blood platelets was chosen for two main reasons. First, oxidative stress plays important role platelet activation. Second, as a part of the haemostatic system, blood platelets may undergo oxidative and nitrative modifications induced by external reactive oxygen species (ROS), generated in the cardiovascular system. The first report suggesting the possibility of ROS generation in platelets was published in 1977 [30]. Since then, it has been found that oxidative stress influence functions of various elements of the haemostatic system, including modulation of platelet activity [31–33], changes in fibrinogen polymerization [34, 35] as well as impairment of fibrinolysis [36, 37]. Activation of blood platelets initiates conformational changes in platelet membrane receptors and numerous intra-platelet processes such as exocytosis of granules, secretion of vasoactive mediators, and cytoskeleton reorganization. It is known that immune mediators and ROS released from platelets may participate in the initiation and maintaining of vascular inflammation [38]. Moreover, ROS generated by inflammatory cells or by activated blood platelets, promote oxidative stress and modulate platelet functions [39, 40]. Excessive production of some oxidants (${\text{O}}_{2}^{ \bullet - }$, for instance) seems to be particularly important in the pathogenesis of cardiovascular disorders. The rapid reaction of NO^∙^ with ${\text{O}}_{2}^{ \bullet - }$ decreases bioavailability of nitric oxide and generates another oxidative factor - peroxynitrite (ONOO^−^) [41]. The role of oxidative stress in platelet activation may be also indirectly confirmed by results from research on different antioxidants. For example, platelet hyperaggregability, accompanied by high intraplatelet production of oxidants, was found by Monteiro et al. [42] in studies on animal model of atherosclerosis. The use of antioxidants such as polyethylene glycol-conjugated catalase (PEG-catalase) and *N*-acetylcysteine effectively prevented hyperactivation of blood platelets. According to Davì et al. [43], in humans, a daily dosage of 100–600 mg, vitamin E significantly decreases the urinary excretion of 11-dehydro-thromboxane B_2_, a marker of platelet activation. Furthermore, beneficial effects of consumption of flavonoid-rich dark chocolate (50 g/day) on oxidative stress, including peroxynitrite generation in platelets, was demonstrated. Peroxynitrite generation was reduced in women by 24.0 % and in men by 18.6 %, whereas NO release was increased by 15.7 % in women, and by 32.2 % in men [44].

Antioxidant action of phenolic fractions of *T. pratense*, *T. scabrum*, and *T. pallidum* was found in our earlier studies [45]. In this work, we extended our examination of biological actions of *Trifolium* plants and analyzed 6 other clover species. For induction of oxidative stress, we used 100 μM peroxynitrite, a strong oxidative and nitrative species, which is generated in the cardiovascular system in vivo. Despite its short half-life (less than 1 s), ONOO^−^ can traverse a mean distance of 3.0, 5.5, and 0.5 µm in mitochondria, blood plasma, and erythrocytes, respectively [46]. The ability of plant polyphenols to deactivate ONOO^−^ is mainly attributed to the presence of hydroxyl groups in their structure, particularly the aromatic –OH groups. In flavonoid molecule, the –OH group at 3 position in the C ring is crucial for ONOO^−^ scavenging. The antioxidant action of this group is enhanced by another –OH groups, localized at positions 5 and 7 as well as by the double-bonded oxygen at position 4 and the ring oxygen at position 1 [47]. From the physiological point of view, only the nano- and micromolar concentrations (less than 5 µg/ml) of phenolic substances are likely to occur in blood plasma in vivo [48]. However, biological activity of most clover species has not been described yet. Hence, we put our attention on the informative aspect of the obtained results. Antioxidant effects of the examined extracts were assessed and compared in a relatively wide concentration range (1.5–50.0 µg/ml), which has been established during our previous experiments. The lowest concentrations of the extracts used in our studies (1.5 µg/ml) may correspond to the range of physiological level of plant-derived phenolic compounds, detected after oral consumption of products rich in polyphenols or dietary supplements. For instance, it has been reported that a single dose of commercial, red clover-based dietary supplement, containing 38.8 mg of isoflavones, results in following plasma concentrations of metabolites: 0.35 μM for irilone, 0.39 μM for daidzein, and 0.06 μM for genistein [49]. In other studies, a single oral bolus dose of 50 mg of either genistein or daidzein, resulted in plasma their concentrations about 800 ng/ml, corresponding to 3.0 µM for genistein and 3.2 µM for daidzein, respectively [50].

Our work revealed that *T. alexandrinum* extract was most effective antioxidatively. Its highly effective action was considerably stronger in measurements of 3-NT, thiol groups, and TBARS. Immunodetections of 3-NT, performed by the WB method demonstrated no chemiluminescence signal for samples preincubated with this extract. On the other hand, the c-ELISA test indicated some concentration of 3-NT in samples of blood platelets and plasma preincubated with *T*. *alexandrinum*. Similar effect was found for (−)-epicatechin. Most likely, it is a result of significant differences in sensitivity of the used analytical methods. Besides the mentioned divergence, WB tests confirmed differences in antioxidant actions of individual extracts. Antioxidant activities of *T. hybridum*, *T. fragiferum*, and *T. resupinatum* var. *resupinatum* are slightly less effective, particularly in prevention of thiol groups oxidation. These observations are partly consistent with our previous results, derived from measurements of free radical scavenging and reducing abilities of the examined extracts [19]. *T. alexandrinum* and *T. incarnatum* extracts contained about 2–3 time higher concentrations of phenolics (52.55 and 47.97 mg/g of dry mass, respectively), compared to the remaining four species. In our previous experiments, *T.alexandrinum* preparation was the most effective scavenger of ABTS and DPPH radicals, followed by *T. resupinatum* var. *resupinatum* and *T. resupinatum* var. *majus* extracts, whereas *T. incarnatum*, *T. hybridum* and *T. fragiferum* were significantly weaker. Extracts of *T. fragiferum*, *T. hybridum*, *T. resupinatum* var. *majus* and *T. resupinatum* var. *resupinatum* contained lower concentrations of polyphenolics - 11.30, 15.24, 22.54, and 17.32 mg/g of dry mass, respectively. Compared to promising results from previous radical scavenging assays [19], during the present study, antioxidant actions of *T. resupinatum* var. *majus* and *T. resupinatum* var. *resupinatum* were less effective.

The fact that the antioxidant capacities of several polyphenolics are not additive in biological systems has been already described in literature [51]. Since the total content of phenolic substances in the individual *Trifolium* extracts does not strictly translates to biological effects of these preparations, differences in antioxidant their actions may be consequence of phytochemical compositions. At this stage of studies, is not possible to undoubtedly indicate which of the phenolics that are present in the examined clover species predominantly contribute to their antioxidative properties; however, two groups of compounds seem to be the most important for this effect: clovamides and isoflavones. In comparison to *T. alexandrinum*, the *T. incarnatum* extract has no clovamides and over three times lower content of isoflavones, but about two times higher concentration of other flavonoids. During assays such TBARS measurements and 3-NT immunodetections, effects of *T. incarnatum* were comparable to other four *Trifolium* extracts, containing considerably lower concentrations of polyphenolic substances. These findings suggest that high biological activity of *T. alexandrinum* may be attributed to the presence of clovamides (9.63 mg/g of dry mass) as well as to high concentration of isoflavones (18.97 mg/g of dry mass). Clovamides, a group of caffeic acid esters, have been found to effectively reduce harmful effects of oxidative stress, mainly due to the ability to prevent lipid peroxidation [52]. Sanbongi et al. [53] demonstrated that clovamide might be more effective antioxidant than well-known antioxidants - epicatechin and quercetin. Furthermore, in our previous work, we demonstrated that the clovamide fraction from *T. pallidum* displayed antioxidant properties and it might partly protected not only lipid, but also protein components of blood platelets and plasma [54]. The isoflavone component of *T.alexandrinum* may additionally enhance antioxidant action of this extract. It has been shown that these compounds may act as antioxidants in vitro and in vivo [55–59].

The available evidence of therapeutic effects of preparations based of clovers other than *T. pratense* have originated mainly from traditional medicine. In this work we compare, for the first time, antioxidant efficacy of plant extracts from six *Trifolium* species in the protection of blood platelets. Our results demonstrate that the examined extracts may partly protect protein and lipid component of blood platelets against ONOO^−^-induced damage. The extract of *T. alexandrinum* seems to be the most potent antioxidant. However, the influence of both this and other *Trifolium* extracts on blood platelets are inadequately known. Therefore, the further studies are needed.
